# Alcohol drinking as an unfavorable prognostic factor for male patients with nasopharyngeal carcinoma

**DOI:** 10.1038/srep19290

**Published:** 2016-01-18

**Authors:** Yu-Pei Chen, Bing-Cheng Zhao, Chen Chen, Xin-Xing Lei, Lu-Jun Shen, Gang Chen, Fang Yan, Guan-Nan Wang, Han Chen, Yi-Quan Jiang, Yun-Fei Xia

**Affiliations:** 1 Department of Radiation Oncology, Sun Yat-sen University Cancer Center, State Key Laboratory of Oncology in South China, Collaborative Innovation Center of Cancer Medicine, Guangzhou, Guangdong 510060, P. R. China; 2 Sun Yat-sen University Cancer Center, State Key Laboratory of Oncology in South China, Collaborative Innovation Center of Cancer Medicine, Guangzhou, Guangdong 510060, P. R. China; 3 Zhongshan School of Medicine, Sun Yat-sen University, Guangzhou, Guangdong 510080, P. R. China

## Abstract

The relationship between alcohol drinking and the prognosis of nasopharyngeal carcinoma (NPC) is unknown. To investigate the prognostic value of alcohol drinking on NPC, this retrospective study was conducted on 1923 male NPC patients. Patients were classified as current, former and non-drinkers according to their drinking status. Furthermore, they were categorized as heavy drinkers and mild/none drinkers based on the intensity and duration of alcohol drinking. Survival outcomes were compared using Kaplan–Meier analysis and Cox proportional hazards model. We found that current drinkers had significantly lower overall survival (OS) rate (5-year OS: 70.2% vs. 76.4%, *P* < 0.001) and locoregional recurrence-free survival (LRFS) rate (5-year LRFS: 69.3% vs. 77.5%, *P* < 0.001) compared with non-drinkers. Drinking ≥14 drinks/week, and drinking ≥20 years were both independent unfavorable prognostic factors for OS (hazard ratio [HR] = 1.38, 95% confidence interval [CI] 1.05–1.81, *P* = 0.022; HR = 1.38, 95% CI 1.09–1.75, *P* = 0.007). Stratified analyses further revealed that the negative impacts of alcohol were manifested mainly among older patients and among smokers. In conclusion, alcohol drinking is a useful predictor of prognosis in male NPC patients; drinkers, especially heavy drinkers have poorer prognosis.

Nasopharyngeal carcinoma (NPC) is a unique head and neck cancer (HNC) endemic in southeast Asia, and is among the most common causes of cancer mortality in China^1^. Radiotherapy (RT) has become the mainstay of treatment, and chemoradiotherapy (CRT) is considered for patients with advanced stage tumors that have been shown to have a relatively poor prognosis when treated with RT alone^2^. However, as lots of factors can affect the biological behavior of tumor as well as the condition of patient, the tumor stage alone is not good enough for survival prediction and treatment planning^3^. Thus the identification of these prognostic factors may assist in the individually tailored treatment for NPC.

Lifestyle factors, such as tobacco smoking and alcohol drinking, are established risk factors for the development of NPC^4^,^5^. It is reasonable to suspect that these lifestyle factors might also affect the prognosis of NPC patients. We have discussed in details the prognostic significance of smoking on NPC in a previous work^6^. The association between alcohol drinking and NPC prognosis remains poorly understood.

There have been a number of studies on the prognostic value of alcohol intake on HNC^7^,^8^,^9^,^10^,^11^,^12^,^13^,^14^,^15^. Park *et al*.^13^ studied a cohort of 580 patients, and found that patients who drunk at least 124.2 g alcohol per day had a significantly elevated death rate as compared with the non-drinkers. Dikshit’s study^9^ of 931 patients also supported that heavy drinking (>121 g/day) could worsen the prognosis of HNC. However, reports on the specific prognostic value of alcohol drinking on NPC patients are few and far from concrete^16^,^17^.

In order to provide additional evidence of the impact of alcohol intake on NPC, we reviewed a large database of NPC patients in our hospital. Besides comparing drinkers (former and current) with non-drinkers, we also categorized patients according to intensity and duration of alcohol consumption to further analyze the associations between drinking and prognosis of NPC. Since female drinkers were few in our database, only male NPC patients were taken into analysis to avoid the confounding effect of gender.

## Materials and Methods

### Patient selection and clinical staging

A retrospective review was conducted of NPC patients treated at Sun Yat-sen University Cancer Center (SYSUCC) between January 2001 and December 2004. Inclusion criteria were: (1) newly diagnosed, histologically proven NPC; (2) without distant metastasis; (3) receiving radical radiotherapy; (4) male patients. A total of 2008 patients were identified. Exclusion criteria were: (1) lack of the record of alcohol intake habits (n = 72); (2) patients younger than 18 years old (n = 13). Thus, the remaining 1923 male NPC patients were enrolled in this study. Computed tomography (CT) and/or magnetic resonance imaging (MRI) was essential for disease staging before treatment, and all patients were restaged according to the 7th edition of the Union for International Cancer Control/American Joint Committee on Cancer (UICC/AJCC) staging system for NPC^18^.

### Data collection

This study was approved by the Institutional Review Board of SYSUCC, and was conducted in accordance with the guidelines of our institution. As this was a retrospective analysis of routine data, we were granted a waiver of written consent, and verbal informed consent was obtained from the patients. The information of patients’ alcohol intake, including drinking status, amount of alcoholic beverages intake per month, duration and quitting time, was collected by physicians at entry and by nurses during hospitalization at SYSUCC. Only the records consistent between both physician and nurse were considered credible. The amount of alcoholic beverages intake was initially recorded in jin, a traditional Chinese unit of weight which equals to 500 grams, and was converted into ounces (one jin equals to 17.64 ounces) later. In our institution, the information on types of alcoholic beverages intake is not necessarily registered on admission; not all medical records collected this information. For the 2008 patients identified initially in our study, there were 210 cases that we needed to collected information on types of alcoholic beverages through telephone follow-up. The telephone follow-up was conducted in June 2014. 72 patients couldn’t be reached for inquiry. These patients were excluded (as mentioned above in the Exclusion criteria). If patients did not drink certain type of alcoholic beverages regularly, it was defined as random type. A medium serving size (one drink) was defined as 12 ounces of beer, 6 ounces of wine, 1.5 ounces of liquor, and an average amount, 6.5 ounces, of random type^19^.

Patients who had consumed alcoholic beverages at least once a week for a minimun of 1 year were categorized as drinkers and the rest as non-drinkers. Drinkers were further categorized as former drinkers who quitted drinking for more than six months and the rest as current drinkers. Quantitative analysis of alcohol intake was evaluated by the intensity and duration of alcohol consumption. The intensity of alcohol intake was defined in terms of drinks per week (drinks/week). Patients with an intensity of ≥14 drinks/week or a duration of ≥20 years were divided as heavy drinkers, and the rest as mild/non drinkers.

As smoking is strongly related to drinking, the status of smoking was also considered in this study. The information on smoking habits of patients, similar to that on alcohol intake habits, was complete and non-smokers were identified as those who never smoked and the rest as smokers^6^,^20^. The quantity of smoking was evaluated by smoking index, which was calculated by multiplying cigarette packs per day and years; NPC patients were divided into low (<15 pack-years) and high (≥15 pack-years) degrees of smoking index groups^6^.

### Treatment

Details of the RT techniques used at SYSUCC were described previously^6^,^21^. Neoadjuvant or adjuvant chemotherapy consisted of cisplatin with 5-fluorouracil or taxanes every three weeks for two or three cycles. Concurrent chemotherapy consisted of either cisplatin plus 5- fluorouracil or cisplatin alone given weekly or on weeks 1, 4 and 7 of radiotherapy. Reasons for incompliance included refusal by individual patients or age or organ dysfunction suggestive of intolerance to treatment.

### Follow-up

After completion of treatment, patients were followed up every 3 months for the first 3 years and the intervals gradually increased to 6–12 months after 3 years. The follow-up duration was calculated from the first day of treatment to the day of death or the last examination.

The primary end point was overall survival (OS), and the secondary end point was locoregional recurrence-free survival (LRFS) and distant metastasis free-survival (DMFS). OS was calculated as time from start of treatment to death from any causes. LRFS and DMFS were calculated as time from start of treatment to the first occurrence of locoregional or distant failure, respectively.

### Statistical analysis

All analyses were performed using SPSS software, version 19.0. We compared the categorical variables in different groups using the Chi-square test. The rates of OS, LRFS and DMFS were estimated by means of the Kaplan-Meier method and differences were compared using the log-rank test. Multivariate analysis using a Cox proportional hazards model was used to test the independent significance of different variables by enter method of insignificant explanatory variables. The covariates entering into the multivariable analysis included host factors (age), tumor factors (T and N classification), treatment factors (chemo-RT or RT), smoking status, and smoking index.

## Results

### Patient characteristics

The baseline characteristics of the whole patients analyzed in this study are shown in Table 1. The median age was 46 years (range, 18–78 years). Among the 1923 patients, 1736 (90.3%) had undifferentiated non-keratinizing carcinoma, 174 (9.0%) had differentiated non-keratinizing carcinoma and 13 (0.7%) had other types. All patients were treated with definitive-intent radiotherapy, with 1753 (91.2%) patients treated with two-dimensional conformal radiotherapy (2D-CRT), 45 (2.3%) patients treated with three-dimensional conformal radiotherapy (3D-CRT) and 125 (6.5%) patients treated with IMRT. 1038 (54%) of 1923 patients received neoadjuvant, concurrent, or adjuvant chemotherapy. The majority of the patients (821 of 1170; 70.2%) with stage III or IV NPC (classified as T3–T4 and/or N2–N3 disease) received chemotherapy.

The median duration of follow-up was 80.5 months (range, 1.6–124.6 months). Up to the last day of follow-up, 579 (30.1%) of the 1923 patients developed locoregional failure, 368 (14.0%) developed distant metastasis and 593 (30.8%) died. For the entire cohort, the 5-year OS, LRFS and DMFS rates were 75.0%, 75.7% and 85.6%, respectively.

In total, 364 patients (18.8%) were drinkers, and drinking status had a strong and significant correlation with age and smoking status. Drinkers, as compared with non-drinkers, had a significantly higher percentage of older (≥47 years) patients (62.9% vs. 46.4%; *P* < 0.001) and smokers (89.8% vs. 51.5%; *P* < 0.001) (Table 1). T classification (T1-T4) was significantly correlated with drinking status (*P* = 0.023), but when dividing T classification into early (T1-2) and advanced (T3-4) ones, no significant correlation was found (*P* = 0.535). Among the 364 drinkers, the types of alcoholic beverages were 6 (1.6%) for beer, 9 (2.5%) for wine, 195 (53.6%) for liquor and 154 (42.3%) for random type. Detail information on alcohol intake in the 364 male NPC drinkers are shown in Supplementary Table S1.

### Prognostic value of status of alcohol intake in male NPC patients

**Figure 1:**
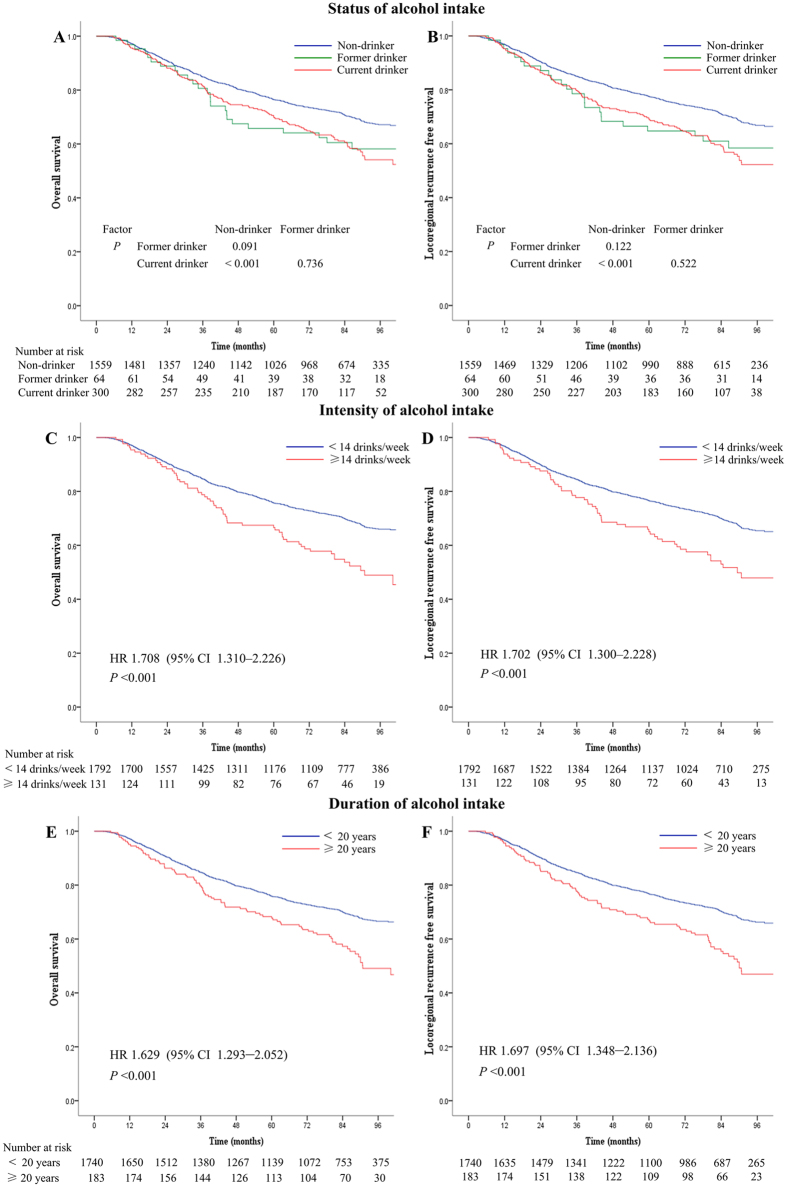
Kaplan-Meier survival curves are shown for (A,C,E) overall survival and (B,D,F) locoregional recurrence free survival in male nasopharyngeal carcinoma patients in survival analysis of (**A,B**) status of alcohol intake, (**C,D**) intensity of alcohol intake and (**E,F**) duration of alcohol intake. Hazards ratios (HRs) were calculated using the unadjusted Cox proportional hazards model. *P*-values were calculated using the unadjusted log-rank test.

In the 364 drinkers, 64 (17.6%) were former drinkers while 300 (82.4%) were current drinkers. Between non-drinker, former drinker and current drinker, significant differences were found in 5-year OS rate (76.4%, 65.7% and 70.2%) and 5-year LRFS rate (77.5%, 64.6% and 69.3%) (All *P* < 0.001) (Fig. 1A,B), while no significant difference was found in 5-year DMFS rate (85.8%, 79.0% and 85.3%; *P* = 0.577). As compared with non-drinkers, the risks of death and locoregional recurrence were significantly higher for current drinkers (All *P* < 0.001) (Fig. 1A,B), and the former drinker had a tendency to have increased risk of death and locoregional recurrence though no significant differences were found (Fig. 1). In the multivariate analysis, current drinking were found to be an independent unfavorable prognostic factors for OS (hazard ratio [HR] = 1.24, 95% confidence interval [CI] 1.01–1.53, *P* = 0.043) and LRFS (HR = 1.30, 95% CI 1.06–1.60, *P* = 0.013) (Table 2). No significant differences existed between former and current drinkers for all outcomes in both univariate and multivariate analyses.

### Prognostic value of intensity of alcohol intake in male NPC patients

In all 1923 patients, 131 (6.8%) drank ≥14 drinks/week and 1792 (93.2%) drank <14 drinks/week. The 5-year OS rate (66.5% vs. 75.7%; *P* < 0.001) and LRFS rate (65.1% vs. 76.6%; *P* < 0.001) for heavy drinkers (≥14 drinks/week) were significantly lower than the corresponding rates for mild/none drinkers (<14 drinks/week) (Fig. 1C,D). No significant difference was found in 5-year DMFS rate (84.2% vs. 85.6%; *P* = 0.316). In the multivariate analysis, drinking ≥14 drinks/week was found to be an independent unfavorable prognostic factors for both OS (HR = 1.47, 95% CI 1.12–1.92, *P* = 0.006) and LRFS (HR = 1.39, 95% CI 1.05–1.84, *P* = 0.023) (Table 2). No significant differences existed between mild (0–14 drinks/week) and none drinkers for both OS and LRFS (Supplementary Fig. S1).

### Prognostic value of duration of alcohol intake in male NPC patients

In all 1923 patients, 183 (9.5%) drank ≥20 years and 1740 (90.5%) drank <20 years. The 5-year OS rate (68.3% vs. 75.8%; *P* < 0.001) and LRFS rate (67.3% vs. 76.7%; *P* < 0.001) for heavy drinkers (≥20 years) were significantly lower than the corresponding rates for mild/none drinkers (<20 years) (Fig. 1E,F). No significant difference was found in 5-year DMFS rate (86.3% vs. 85.4%; *P* = 0.991). In the multivariate analysis, drinking ≥20 years was found to be an independent unfavorable prognostic factors for both OS (HR = 1.30, 95% CI 1.02–1.66, *P* = 0.037) and LRFS (HR = 1.36, 95% CI 1.07–1.77, *P* = 0.013) (Table 2). No significant differences existed between mild (0–20 years) and none drinkers for both OS and LRFS (Supplementary Fig. S1).

### Prognostic value of alcohol intake in male NPC patients stratified by age and smoking status

As drinking was significantly correlated with age (≥47 vs.<47 years), and smoking status (smoker vs. non-smoker), we analyzed the prognostic value of alcohol intake in male NPC patients stratified by age and smoking status, respectively.

In the univariate analysis, current drinkers had significantly unfavorable OS and LRFS when compared with non-drinker, in both patients ≥47 years (*P* = 0.018, *P* = 0.008), and patients <47 years (*P* = 0.032, *P* = 0.004). Patients drinking ≥20 years also had significantly unfavorable OS and LRFS in patients ≥47 years (*P* = 0.030, *P* = 0.012), and patients <47 years (*P* = 0.047, *P* = 0.010), while patients drinking ≥14 drinks/week only had significantly unfavorable OS and LRFS in patients ≥47 years (*P* = 0.003, *P* = 0.002). Table 2 presents the results of the multivariate analysis in patients stratified by age. Current drinking, drinking ≥14 drinks/week, and drinking ≥20 years were independent unfavorable prognostic factors for OS and LRFS only in patients ≥47 years (Table 2).

For patients stratified by smoking status, univariate analysis indicated that when compared with non-drinkers, current drinkers had unfavorable LRFS in both smokers and non-smokers (*P* = 0.008, *P* = 0.009), while had unfavorable OS only in smokers (*P* = 0.011). Patients drinking ≥14 drinks/week had significantly unfavorable OS and LRFS only in smokers (*P* = 0.002, *P* = 0.001), and patients drinking ≥20 years had significantly unfavorable OS and LRFS in both smokers (*P* = 0.005, *P* = 0.005) and non-smokers (*P* = 0.018, *P* = 0.001). Table 2 presents the results of the multivariate analysis in patients stratified by smoking status. Current drinking, and drinking ≥14 drinks/week were independent unfavorable prognostic factors for OS and LRFS in smokers, while drinking ≥20 years was an independent unfavorable prognostic factor for LRFS in non-smokers and smokers (Table 2).

## Discussion

Some lifestyle behaviors are established risk factors and potential prognostic factors for NPC. In our study, we examined the association of alcohol intake with the survival of male NPC treated with RT. The results showed that current alcohol drinking predicted a poorer prognosis, and that former alcohol drinking had a tendency to increase risk of death and recurrence, though without significance. The negative impact of alcohol on prognosis was mainly caused by alcohol intake with an intensity of ≥14 drinks/week or a duration of ≥20 years, while no significant survival difference was noted between mild and none drinkers (an intensity of 0–14 drinks/week or a duration of 0–20 years). Stratified analyses further revealed that the negative impacts of alcohol were manifested mainly among older patients and among smokers.

Many reports have discussed the prognostic significance of alcohol drinking for HNC. In an article by Broglie *et al*.^8^ in which a risk-of-death categories model was established for oropharyngeal cancer, alcohol consumption was a major predictor of OS, only following HPV status, and the impact of alcohol on outcome was even more pronounced than that of smoking. However, there are few systematic studies on the association between alcohol drinking and NPC so far. Only two studies mentioned the impact of drinking on NPC^16^,^17^. In Ji’s cohort of 276 patients with stage II-IVb NPC treated by IMRT ± chemotherapy, alcohol consumption was a negative prognostic factor for OS, with *P* < 0.001 in univariate analysis and *P* = 0.06 in multivariate analysis^16^. Shen *et al*. also noted the associations between the frequency (>1 drink/day) and duration (≥20 years) of alcohol drinking and poor survival of NPC patients in univariate analysis (*P* = 0.001 and *P* = 0.019, respectively), but both lost significance after adjustment for clinical variables and demographic characteristics^17^. In our study, the prognostic value of drinking in male NPC patients was discussed in details. We found that drinkers, especially heavy drinkers with a high intensity and/or quantity of alcohol consumption, had poorer OS, and the conclusions remained valid after adjustment for potential confounding variables.

Current drinkers were found to have significantly inferior OS and LRFS when compared with non-drinkers, while no significant differences in OS and LRFS were detected between former drinkers and non-drinkers. However, the Kaplan-Meier survival curves of former and current drinkers are generally overlapped, and former drinkers had a tendency to increase risk of death and recurrence when compared with non-drinkers (*P* = 0.091, *P* = 0.122) (Fig. 1C,D). The insignificance of differences between former and non-drinkers was probably due to the small number of former drinkers. Former and current drinking might have similar impact on prognosis of NPC patients. A large sample size retrospective study conducted in HNCs patients showed that the risk of local failure was 1.3 fold higher in active drinkers and 1.15 fold higher in former drinkers, compared with never drinkers; the risk of death was 1.27 fold higher in active drinkers and 1.1 fold higher in former drinkers compared with never drinkers^15^. But no significant difference was found between active and former drinkers for both outcome. In our study, the HRs for OS and LRFS of former drinkers (HR = 1.19, HR = 1.15) were relatively smaller than those of current drinkers (HR = 1.24, HR = 1.30); but no significant differences were found between former and current drinkers for OS and LRFS in our study. When compared with current drinking, whether abstinence might bring little benefit for alcohol drinkers in NPC patients need further researches. Larger prospective studies are needed to clarify the prognostic significance of different drinking status.

Why alcohol drinkers with NPC had unfavorable prognosis ? Besides the increased possibility of smoking in this group of people^11^, which was an established negative prognostic factor for NPC and had been adjusted in our study, other explanations have been proposed. First of all, alcoholics tends to consume less foods with essential nutrients, and alcohol and its metabolites may further disrupt the absorbing and using of those nutrients, leading to malnutrition of the body^22^,^23^. The study of Nunez *et al*. showed that ethanol consumption can invoke a strong depletion of body fat, facilitate wasting and shorten survival time of tumor-bearing mice^24^. Second, alcohol consumption can compromise the immune surveillance and clearance function, resulting from a combination of altered cytokine production, abnormal reactive oxygen species generation, suppressed natural killer (NK) cells activity and impaired cell-mediated immunity^25^,^26^. For example, Wu *et al*.^27^ suggested that the direct effects of alcohol, together with the deregulated neuroendocrine mediators glucocorticoids and catecholamines were the cause of splenic NK cell suppression in a mouse binge drinking model. Third, microRNA expression in HNC was also found to be altered with alcohol consumption, and the high expression of miR-21 was associated with significantly decreased 5-year survival^7^. Other factors leading to the worse outcomes among alcohol drinkers may include worse response rates to chemotherapy, smaller radiation doses delivered, and noncompliance^28^. We found that the unfavorable prognosis of alcohol drinkers was mainly observed among the elders and smokers. This is probably explained by fact that older patients were in poorer health condition and were more prone to negative impact of alcohol. The number of drinkers among non-smokers was so small in this study that the analyses were underpowered, which might result in the insignificant prognostic effects of alcohol drinking in the non-smokers.

In this study, we reviewed a large cohort of male NPC patients (n = 2008) from a single institute, and enrolled 1923 of them for analysis. To the best of our knowledge, this is the first report about the significant impact of status, intensity and duration of alcohol drinking on the prognosis of NPC. The limitations of our study are related to its retrospective nature. We found it difficult to obtain the exact amount of alcohol consumption, since most people drink more than one kind of alcoholic beverage with different alcohol proofs. Different kinds of alcoholic beverage (such as wine and liquor) may have opposite effects on the prognosis of malignancies^29^. These may lead to potential bias. Besides, the drinking habit of patients after treatment, which is usually abstinence from alcohol, was not considered in the present study. This may have an impact on prognosis as well. Another limitation is that the number of drinkers among non-smokers was small (only 37) in our cohort, so the prognostic value of alcohol drinking for this group of patients needs assurance through further research. Finally, though unhealthy diet, poor health conditions, and social dysfunction related to alcohol drinking may explain the worse prognosis of alcohol drinkers, their mediating effect could not be confirmed in this retrospective study because of lack of relevant data, and future prospective studies are needed.

Nevertheless, this study adds to evidences supporting the unfavorable impact of heavy drinking on the prognosis of NPC patients. Further studies are required to assure the prognostic effect of alcohol drinking, and establish optimal therapeutic regimens for these patients.

## Additional Information

**How to cite this article**: Chen, Y.-P. *et al*. Alcohol drinking as an unfavorable prognostic factor for male patients with nasopharyngeal carcinoma. *Sci. Rep.* **6**, 19290; doi: 10.1038/srep19290 (2016).

## Supplementary Material

Supplementary Information

## Figures and Tables

**Table 1:** Baseline characterictics of the 1923 male NPC patients (non-drinker vs. drinker).

Patient characteristics	Non-drinker (*N *= 1559) *n* (%)	Drinker (*N *= 364) *n* (%)	*P*-value
Age (years)			
<47	836 (53.6)	135 (37.1)	<0.001
≥47	723 (46.4)	229 (62.9)	
Clinical stage^a^			
I	94 (6.0)	18 (4.9)	0.751
II	521 (33.4)	120 (33.0)	
III	612 (39.3)	152 (41.8)	
IV	332 (21.3)	74 (20.3)	
T classification^a^			
T1	323 (20.7)	55 (15.1)	0.023
T2	570 (36.6)	160 (44.0)	
T3	385 (24.7)	90 (24.7)	
T4	281 (18.0)	59 (16.2)	
N classification^a^			
N0	406 (26.0)	85 (23.4)	0.457
N1	637 (40.9)	143 (39.3)	
N2	456 (29.2)	120 (33.0)	
N3	60 (3.8)	16 (4.4)	
Treatment			
RT alone	716 (45.9)	169 (46.4)	0.863
CRT	843 (54.1)	195 (53.6)	
Smoking status			
Non-smoker	756 (48.5)	37 (10.2)	<0.001
Smoker	803 (51.5)	327 (89.8)	
Drinking status			–
Former drinker	–	64 (17.6)	
Current drinker	–	300 (82.4)	
Drinking intensity			–
0–14 drinks/week	–	234 (64.3)	
≥14 drinks/week	–	130 (35.7)	
Drinking duration			–
0–20 years	–	181 (49.7)	
≥20 years	–	183 (50.3)	

*Abbreviation:* NPC, nasopharyngeal carcinoma; RT, radiotherapy; CRT, chemoradiotherapy.

^a^According to the 7th edition of the Union for International Cancer Control/American Joint Committee on Cancer staging system.

**Table 2:** Multivariate analyses of alcohol intake variables for all 1923 male nasopharyngeal carcinoma patients and patients stratified by age/smoking status.

Alcohol intake variables	OS		LRFS	
	HR (95% CI)	*P*-value	HR (95% CI)	*P*-value
All patients				
Status of alcohol intake (vs. non-drinker)		0.037	1.15 (0.83–2.32)	0.025
Former drinker	1.19 (0.77–1.79)	0.297	1.15 (0.83–2.32)	0.382
Current drinker	1.24 (1.01–1.53)	0.043	1.30 (1.06–1.60)	0.013
Intensity of alcohol intake (≥14 vs. <14 drinks/week)	1.47 (1.12–1.92)	0.006	1.39 (1.05–1.84)	0.023
Duration effect of alcohol intake (≥20 vs. <20 years)	1.30 (1.02–1.66)	0.037	1.36 (1.07–1.77)	0.013
Patients with an age <47 years				
Status of alcohol intake (vs. non-drinker)		0.624		0.326
Former drinker	0.57 (0.80–4.10)	0.579	0.55 (0.08–3.96)	0.553
Current drinker	1.15 (0.81–1.64)	0.442	1.27 (0.90–1.78)	0.179
Intensity of alcohol intake (≥14 vs. <14 drinks/week)	0.99 (0.44–2.22)	0.978	0.95 (0.42–2.12)	0.897
Duration of alcohol intake (≥20 vs. <20 years)	1.33 (0.76–2.31)	0.314	1.46 (0.86–2.46)	0.161
Patients with an age ≥47 years				
Status of alcohol intake (vs. non-drinker)		0.023		0.029
Former drinker	1.27 (0.98–1.64)	0.059	1.65 (0.99–2.77)	0.055
Current drinker	1.76 (1.07–2.89)	0.026	1.31 (1.02–1.69)	0.039
Intensity of alcohol intake (≥14 vs. <14 drinks/week)	1.61 (1.20–2.15)	0.001	1.53 (1.13–2.08)	0.006
Duration of alcohol intake (≥20 vs. <20 years)	1.31 (1.00–1.70)	0.047	1.34 (1.02–1.76)	0.039
Non-smokers				
Status of alcohol intake (vs. non-drinker)		0.344		0.063
Former drinker	0.96 (0.13–6.88)	0.965	1.15 (0.16–8.27)	0.891
Current drinker	1.55 (0.86–2.78)	0.144	1.93 (1.12–3.35)	0.019
Intensity of alcohol intake (≥14 vs. <14 drinks/week)	1.22 (0.42–3.55)	0.715	1.43 (0.49–4.20)	0.514
Duration of alcohol intake (≥20 vs. <20 years)	1.95 (0.95–3.98	0.069	2.63 (1.33–5.19)	0.005
Smokers				
Status of alcohol intake (vs. non-drinker)		0.058		0.079
Former drinker	1.58 (0.96–2.60)	0.073	1.40 (0.84–2.34)	0.198
Current drinker	1.54 (1.01–2.42)	0.049	1.26 (1.01–1.57)	0.044
Intensity of alcohol intake (≥14 vs. <14 drinks/week)	1.51 (1.14–2.01)	0.005	1.42 (1.20–1.84)	0.020
Duration of alcohol intake (≥20 vs. <20 years)	1.28 (0.99–1.65)	0.061	1.31 (1.01–1.69)	0.041

*Abbreviation:* OS, overall survival; LRFS, locoregional recurrence-free survival; CRT, chemoradiotherapy; RT, radiotherapy; HR, hazard ratio; 95% CI, 95% confidence interval; NS, non-significant.

The following parameters were included in the multivariate analysis using Cox proportional hazards model by enter method: age (≥47 vs. <47 years), T classification (T3–4 vs. T1–2), N classification (N0–1 vs. N2–3), treatment (CRT vs. RT), smoking status (smoker vs. non-smoker), smoking index (≥15 vs. <15 pack-years), and status (former/current drinker vs. non-drinker)/intensity (≥14 vs. <14 drinks/week)/duration (≥20 vs. <20 years) of alcohol intake.
