# Supplementary material for: Alcohol drinking as an unfavorable prognostic factor for male patients with nasopharyngeal carcinoma
Source: Sci Rep. 2016 Jan 18;6:19290. doi: 10.1038/srep19290 (PMC4725964; doi:10.1038/srep19290)
Supplement: Supplementary Information [file srep19290-s1.pdf]

Supplementary information

Title: Alcohol drinking as an unfavorable prognostic factor for male patients with nasopharyngeal carcinoma

Authors list: Yu-Pei Chen, Bing-Cheng Zhao, Chen Chen, Xin-Xing Lei, Lu-Jun Shen, Gang Chen, Fang Yan, Guan-Nan Wang, Han Chen, Yi-Quan Jiang, and Yun-Fei Xia

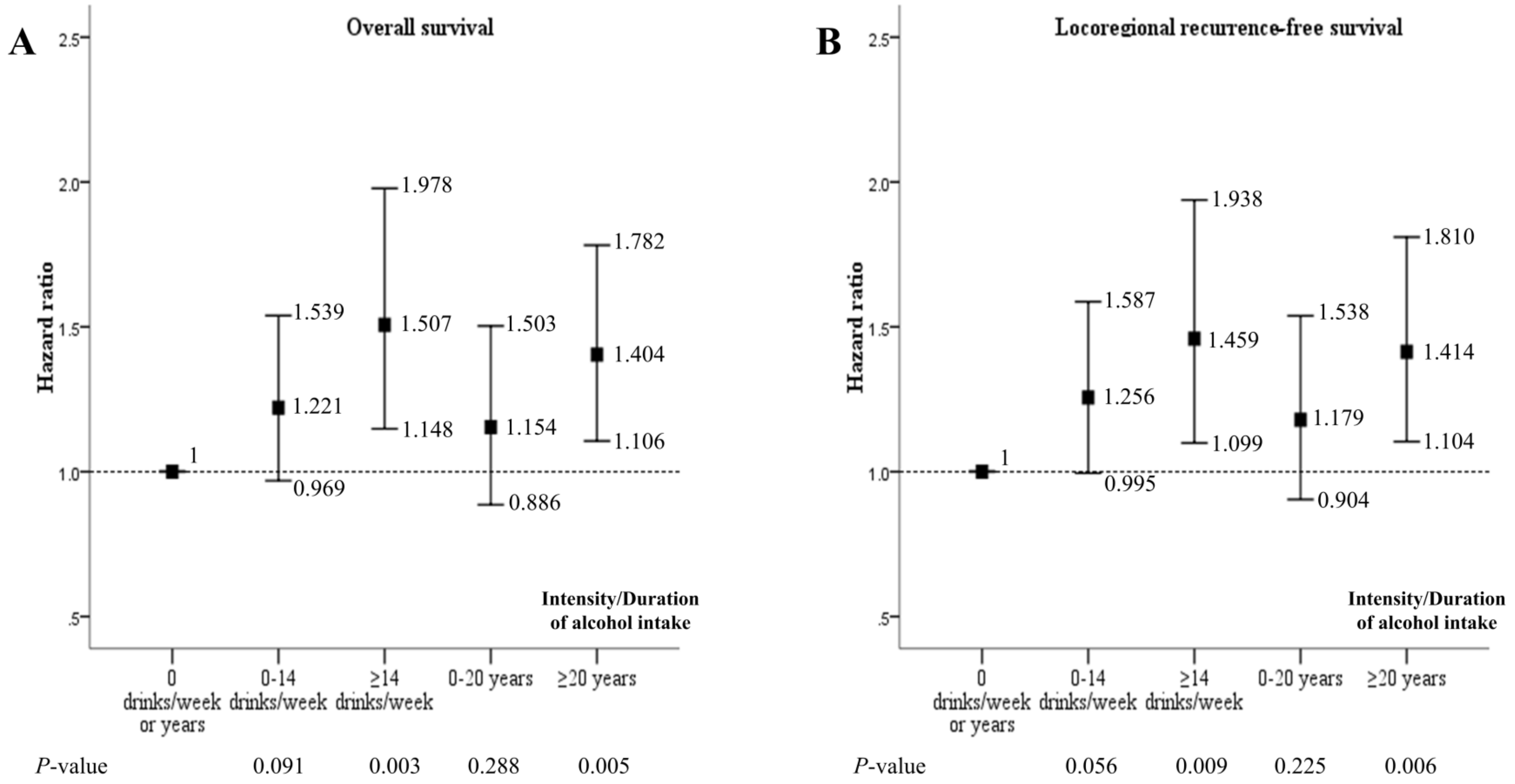

Supplementary Fig. S1. Scatter plot with error bars about hazard ratios (HRs) and the corresponding 95% confidence intervals (95% CIs) of heavy (≥14 drinks/week or ≥20 years), mild (0-14 drinks/week or 0-20 years), and none drinkers are shown for (A) overall survival and (B) locoregional recurrence free survival. HRs and *P*-values were calculated using the adjusted Cox proportional hazards model.

Supplementary Table S1. Detailed information on alcohol intake in the 364 male drinkers with nasopharyngeal carcinoma.

Alcohol intake	Number (%)	Intensity (drinks/week) median (range)	Duration (years) median (range)	Quitting time (years) median (range)
Drinker	364 (100)	7 (1–73)	20 (2–50)	0 (0–15)
Status				
Former drinker	64 (17.6)	9 (1–73)	10 (2–50)	2 (0.5–15)
Current drinker	300 (82.4)	7 (1–59)	20 (2–50)	--
Intensity				
0–14 drinks/week	234 (64.3)	5 (1–12)	10 (2–50)	--
⩾14 drinks/week	130 (35.7)	20 (14–73)	20 (2–40)	--
Duration				
0–20 years	181 (49.7)	6 (1–59)	10 (2–18)	--
⩾20 years	183 (50.3)	10 (1–73)	30 (20–50)	--
